# PitNET tissue deconvolution: tracing normal tissue residues and immune dynamics

**DOI:** 10.3389/fendo.2025.1674625

**Published:** 2025-11-27

**Authors:** Mattia Dalle Nogare, Serena Avallone, Luna Picello, Daniele Puggina, Luca Denaro, Gabriele Sales, Giovanni Vazza, Gianluca Occhi

**Affiliations:** 1 Department of Biology, University of Padova, Padova, Italy; 2 Endocrine Disease Unit, Department of Medicine, University of Padova, Padova, Italy; 3 Department of Neuroscience, University of Padova, Padova, Italy

**Keywords:** pituitary neuroendocrine tumors (PitNETs), bulk RNA sequencing (RNA-seq), single-nucleus RNA sequencing (snRNA-seq), deconvolution methods, cellular heterogeneity

## Abstract

**Background:**

Bulk RNA sequencing (RNA-seq) has substantially advanced the understanding of pituitary neuroendocrine tumors (PitNETs). However, its limited ability to resolve cellular heterogeneity – particularly in samples containing residual non-tumor pituitary cells – remains a significant challenge.

**Objective:**

We developed and validated a tissue deconvolution framework using a reference dataset derived from single-nucleus RNA sequencing (snRNA-seq) of normal pituitary tissue, aimed at estimating cellular composition in PitNETs from bulk RNA-seq data and characterizing the tumor microenvironment (TME).

**Methods:**

Marker-based (CIBERSORT, MuSiC) and single-cell–based (CIBERSORTx, MuSiC) deconvolution approaches were benchmarked across simulated, pseudobulk, and bulk RNA-seq datasets to identify the most reliable tools.

**Results:**

CIBERSORTx demonstrated the highest sensitivity (r > 0.85) for detecting pituitary cell types, although accuracy decreased for TME components. Application to ten GH-secreting PitNETs with known histological contamination and to public datasets consistently revealed residual normal tissue across hormone-secreting subtypes, excluding silent tumors. Contaminated samples – averaging 43% ± 19% with CIBERSORTx and 37% ± 22% with CIBERSORT – displayed distinct transcriptomic profiles compared to uncontaminated, lineage-matched tumors, based on clustering analyses.

**Conclusion:**

This study establishes snRNA-seq–based deconvolution as a robust strategy for reconstructing cellular composition in PitNETs, mitigating the impact of histological contamination and improving the reliability of downstream transcriptomic analyses.

## 1 Introduction

Pituitary neuroendocrine tumors (PitNETs) are benign tumors arising from the anterior pituitary gland. With an annual incidence of 3.9 to 7.4 cases per 100,000 individuals and a clinical prevalence of approximately 1 in 1,000 people (1), PitNETs commonly manifest through hormonal dysregulation or symptoms secondary to mass effect (2). Classification is based on tumor cell lineages, determined through immunohistochemical analysis (IHC) of key transcription factors (SF1, PIT1, TPIT) and pituitary hormone secretion (3). This classification includes PIT1 lineage tumors (lactotroph, somatotroph, thyrotroph, and silent PIT1 tumors), TPIT lineage tumors (corticotroph and silent TPIT tumors), SF1 lineage tumors (gonadotroph and silent SF1 tumors), null cell tumors (lacking both transcription factors and hormone expression), and plurihormonal tumors. Although typically benign, a subset of PitNETs displays aggressive clinical features, including rapid growth, treatment resistance, and invasion of adjacent structures (4). These features highlight the need for deeper molecular characterization to enhance our understanding of PitNET biology and clinical behavior.

Neoplastic transformation is characterized by profound alterations in the transcriptional landscape, reflecting the multistep process driven by oncogene activation and tumor suppressor gene inactivation. This dynamic rewiring contributes to the molecular heterogeneity observed within tumor entities (5–8) and often correlates with diagnostic and prognostic features (9, 10). Transcriptomic analyses have long been central to cancer research, from early genome-wide profiling approaches such as microarrays (5) to more recent next-generation sequencing technologies (11). Pituitary tumors are no exception. Bulk RNA transcriptomic (RNA-seq) studies have been extensively applied to PitNETs, revealing dysregulated molecular pathways and providing insights into tumor heterogeneity (12–14). These studies have often stratified tumors based on clinical and pathological features, including treatment status, invasiveness, and cytokeratin-based granulation patterns (15–17).

Nevertheless, bulk RNA-seq analyses are inherently limited by the composition of surgical specimens. PitNETs frequently infiltrate surrounding healthy tissue, entrapping non-neoplastic cells (18). As a result, even morphologically confirmed tumor samples may be contaminated by non-tumor elements, potentially biasing molecular analyses (19). Sampling core tumor regions – ideally guided by intraoperative collaboration between neurosurgeons and pathologists (12) – can help mitigate this issue, though it is not always feasible in routine clinical practice. Moreover, components of the tumor microenvironment (TME), such as inflammatory infiltrates and stromal fibroblasts, actively shape the bulk transcriptome. Yet their specific contributions remain obscured, adding complexity to data interpretation.

To address these challenges, high-resolution profiling methods such as flow cytometry (20), single-cell RNA sequencing (scRNA-seq) (21), and spatial transcriptomics (22) have been developed, offering unprecedented resolution of the cellular composition within tumors. These technologies have been applied to PitNETs, contributing to a more refined understanding of their cellular heterogeneity (23–25). Despite their transformative potential, however, these approaches remain technically demanding, cost-prohibitive, and impractical for large-scale or routine studies.

As a complementary strategy, computational deconvolution methods have emerged as powerful tools to infer cellular composition directly from bulk gene expression profiles (GEPs) (26). Algorithms such as CIBERSORT (27) and MuSiC (28) leverage either predefined marker genes or reference scRNA-seq datasets to estimate the proportions of distinct cell types within complex tissues. These computational approaches offer a scalable, cost-effective alternative to experimental single-cell techniques. They enable a more nuanced interpretation of bulk transcriptomic data and facilitate the analysis of TME dynamics, even when high-resolution experimental data are unavailable.

Building on these developments, the present study aims to generate a cell-type-specific signature matrix derived from single-nucleus RNA-seq (snRNA-seq) datasets. This matrix will enable robust estimation of cellular composition in PitNET samples analyzed by bulk RNA-seq. As a secondary aim, we seek to leverage this approach to characterize the composition of the TME, providing insights into stromal, immune, and other non-neoplastic components that may influence PitNET biology and clinical behavior.

## 2 Materials and methods

### 2.1 Data sources and code availability

Four normal adult human pituitary samples analyzed by snRNA-seq were obtained from Zhang et al. (GEO: GSE178454) (29). Additionally, 23 PitNETs analyzed by scRNA-seq were retrieved from Yan et al., accessible at http://lifeome.net/supp/pituitary (30). Bulk RNA-seq raw counts were obtained from Neou et al. (E-MTAB-7768) (12) and da Silva-Júnior et al. (GSE209903) (31). All computational codes used in this study are available upon request.

### 2.2 Data preprocessing and quality control

Data preprocessing and quality control were performed in R (v. 4.4.1) using the Seurat package (version 5.2.1) separately for both snRNA-seq and scRNA-seq datasets, following the workflow described below. In the initial step, low-quality cells were filtered out. Only cells with 1,000 < nFeature_RNA < 5,000, nCount_RNA > 200, and mitochondrial RNA content < 5% were retained. Cells predicted as doublets by the DoubletFinder R package (version 2.0.4) were removed.

Pituitary samples were integrated using the merge function, with batch effects corrected via the Harmony algorithm. Principal Component Analysis (PCA) was applied to scaled data to reduce dimensionality and technical noise, retaining the first 30 principal components. Cell clustering was conducted using the FindClusters function, setting resolution = 0.6 for snRNA-seq and 0.5 for scRNA-seq. UMAP embeddings were used for visualization. Cell cluster annotation was based on classical pituitary cell-type markers (29, 32) (see **Supplementary Table 1**), in combination with automated annotation using the SingleR package (version 2.6.0), applying the label.main output and using the HumanPrimaryCellAtlasData as a reference.

### 2.3 Cell type deconvolution algorithms

We employed two distinct strategies to estimate cell-type composition: marker-based deconvolution and snRNA-seq-based deconvolution.

For marker-based methods, we first normalized UMI (unique molecular identifier) counts from processed snRNA-seq data to counts per million (CPM), followed by natural-log transformation. Highly differentially expressed genes (DEGs) were identified across clusters using Seurat’s FindMarkers function (“bimod” method, min.pct = 0.25). DEGs with adjusted p-value ≤ 0.05 and natural-log fold change (logFC) > 1 were considered significant marker. To ensure specificity, we applied a minimum expression threshold of >1 CPM in at least one cell type within the final signature matrix. We then generated gene expression signature (GES) reference matrices by selecting the top 50, 100, 200, and 300 most highly expressed genes per cell type (GES50, GES100, GES150, GES200, GES300; **Supplementary Table 2**). These matrices were used for deconvolution via the CIBERSORT R package (v0.1.0) (27), applying a support vector regression (SVR) algorithm with 100 permutations to assess statistical significance. Deconvolution was also performed with the MuSiC R package (v1.0.0) (28) using the *music_prop* function with *markers* as arguments.

For snRNA-seq-based methods, we applied deconvolution approaches that leverage full snRNA-seq-derived gene expression matrices, avoiding reliance on predefined markers. Specifically, we utilized CIBERSORTx and MuSiC. For CIBERSORTx (33), the full gene expression matrix of single nuclei was analyzed locally using the official Docker image (https://cibersortx.stanford.edu/), where signature matrices were generated internally. The analysis was performed with batch correction set to B-mode, quantile normalization disabled, relative run mode, and 100 permutations. Similarly, in MuSiC, raw expression matrices were used to compute cross-subject-consistent cell-type proportions, following manual parameter settings.

### 2.4 Validation on simulated and PitNET pseudobulk data from snRNA-seq and scRNA-seq

We validated our approach using both simulated and tumor pseudobulk data (see below) derived from scRNA-seq and snRNA-seq datasets respectively. For the simulation, gene expression matrices, normalized to CPM, were extracted from individual cells in the processed snRNA-seq dataset and used to generate pseudobulk profiles with the SimBu R package (v1.6.0). A random mixing scenario was applied, selecting 500 single cells per pseudobulk sample to generate 150 samples, without applying a scaling factor. The simulation produced a pseudobulk expression matrix alongside the corresponding true cellular proportions for each bulk sample. To evaluate the accuracy of the deconvolution algorithms, we performed a Pearson correlation coefficient analysis using the *cor* function in base R, comparing true cellular proportions with the estimated values for each cell type across all software tools. Estimations with p-value < 0.05 and correlation coefficient (r) > 0.70 were considered validated and robust. Deconvolution results were visualized using correlation plots and boxplots, generated with the ggplot2 R package (v3.5.1).

Additionally, we extracted normalized gene expression matrices from 23 PitNET-derived scRNA-seq datasets to validate the method on tumor-origin tissues, ensuring robustness across real biological pseudobulk samples.

### 2.5 Processing and RNA-seq analysis of GH-PitNETs

The study was conducted in accordance with ethical guidelines consistent with the Declaration of Helsinki and was approved by the Ethical Committee of Azienda Ospedaliera di Padova (approval no. AOP1782). 10 GH-PitNETs were collected from active acromegalic patients diagnosed according to current consensus criteria (34) and treated with trans-sphenoidal surgery at Padova University/Hospital. Tissue specimens underwent histopathological processing, including fixation in 10% buffered formalin, paraffin embedding, and hematoxylin/eosin staining for diagnostic evaluation. Immunohistochemical analyses assessed pituitary hormone and transcription factors expression following international guidelines (35). A second fragment from each sample was preserved in RNAlater (Ambion), stored at 4 °C for 24 hours, then maintained at −20 °C until RNA extraction.

Total RNA was extracted using the Quick-DNA/RNA kit (Zymo Research, California, USA), following the manufacturer’s protocol. RNA integrity and yield were assessed using an Agilent 2100 Bioanalyzer (Agilent Technologies, Santa Clara, CA) and a NanoDrop spectrophotometer (NanoDrop Technologies), respectively. DNA contamination was removed using the Turbo DNA-free kit (Ambion).

RNA-seq libraries were prepared using KAPA™ RNA HyperPrep with RiboErase (Roche, Indiana, USA) following manufacturer’s recommendations. Sequencing on a NovaSeq X Plus 25B (Illumina) generated 150 bp paired-end reads. Raw FASTQ files underwent quality control using FastQC (v0.12.1) and trimming of low-quality reads using Trimmomatic (v0.39). Transcript abundances for human genes in ENSEMBL (release 113) were estimated with Salmon (36) and summarized at the gene level using tximport (37). Gene filtering followed expression-based criteria outlined by Chen et al. (38), implemented in edgeR (v4.2.2). Raw data are available upon request from the corresponding author.

### 2.6 Bulk RNA-seq deconvolution validation

To evaluate the performance of the signature in bulk RNA-seq datasets, raw counts from both the in-silico dataset (12, 31) and 10 GH-PitNETs were converted to CPM using the *cpm* function from the edgeR package. Each dataset was processed separately using the best-performing deconvolution software and the optimal signature.

To investigate the expression patterns of specific cell type marker genes (**Supplementary Table 1**) across the 10 GH-PitNET, a heatmap was generated using the pheatmap package (v1.0.12) in R, based on log-transformed expression values log_10_(CPM + 1).

To assess global transcriptomic heterogeneity and evaluate the impact of normal residual tissue on sample distribution, the 10 GH-PitNET samples were integrated with two external datasets and batch-corrected using ComBat from the sva package (v3.54.0). For sample clustering analysis, Euclidean distances were computed on the top 500 most variable genes using the dist() function. Hierarchical clustering was performed with the complete linkage method via the base R function hclust() and visualized using the dendextend package (v1.19.0).

## 3 Results

The results presented below stem from the analytical workflow outlined in **Supplementary Figure 1**, which integrates datasets from both normal pituitary tissue and PitNET. This framework enabled the identification of cell type–specific gene signatures, their validation using simulated bulk profiles, and subsequent deconvolution analyses across multiple PitNET datasets.

### 3.1 Construction of integrated snRNA-seq reference data

**Figure 1:**
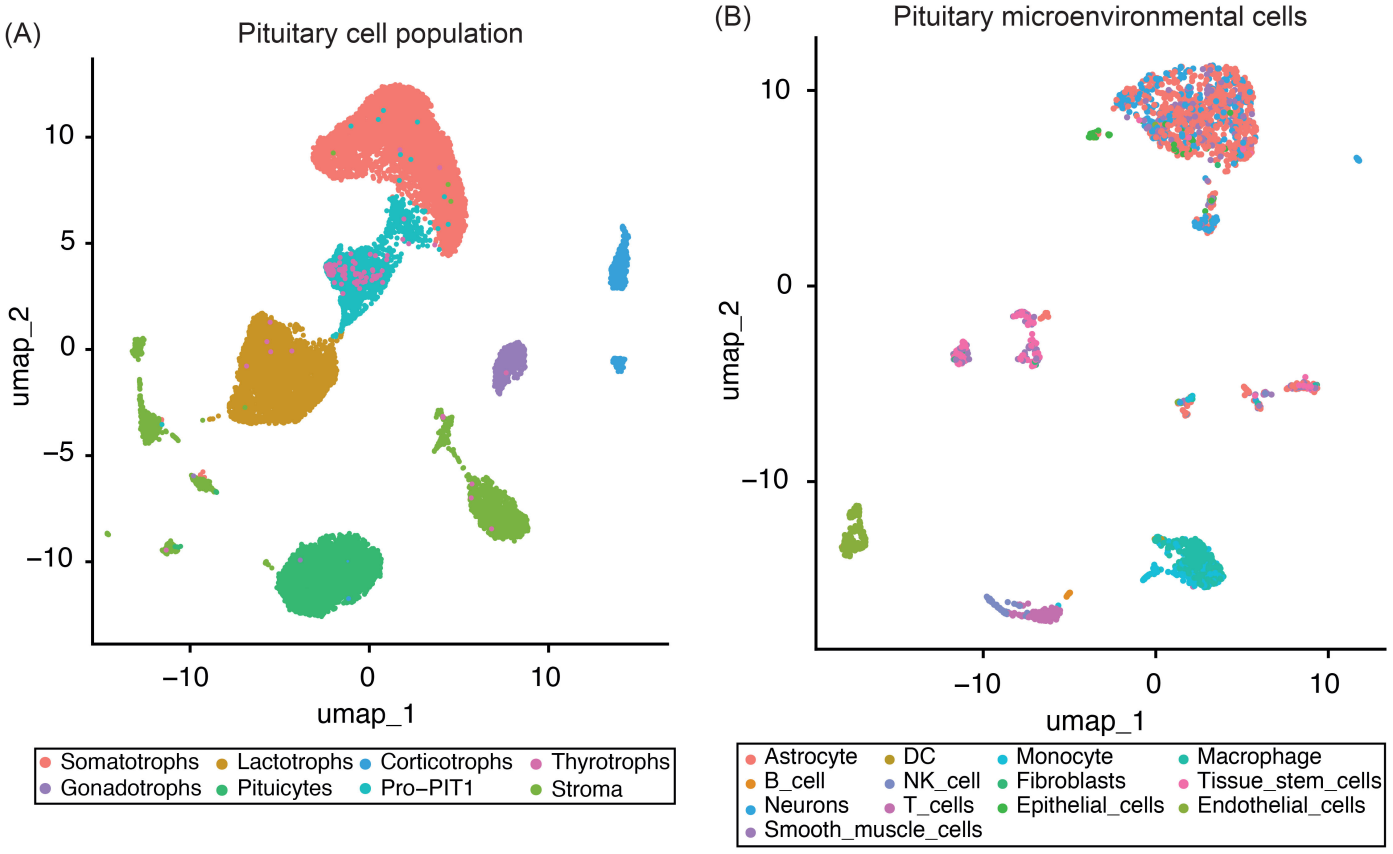
UMAP plots of four normal pituitary snRNA-seq samples. **(A)** UMAP plot showing the integrated single-nucleus RNA-seq data from four normal pituitary samples, with the major pituitary cell populations highlighted. **(B)** UMAP plot displaying only the pituitary microenvironmental cells subset, with distinct stromal and immune cell populations identified using the SingleR package.

To establish a robust reference for deconvolution analyses, we analyzed snRNA-seq data from four normal adult human anterior pituitary samples (two male and two female donors) (29). After applying stringent quality control, normalization, and integration, we retained 18,367 high-quality single-nucleus transcriptomes for downstream analysis. Cell type annotation, performed using canonical marker genes (**Supplementary Table 1**) and the SingleR package, enabled the identification of distinct cell populations within the tissue (**Figure 1**).

Our analysis captured the full spectrum of all major endocrine cell types, including somatotrophs, lactotrophs, thyrotrophs, gonadotrophs, corticotrophs, PIT1-lineage progenitors (Pro-PIT1), and resident stem cells (**Figure 1A**). Non-endocrine populations were categorized into two functionally distinct groups: (i) stromal and extracellular matrix-associated cells – such as smooth muscle cells, endothelial cells, fibroblasts, astrocytes, neurons, and epithelial cells – and (ii) immune cells, including dendritic cells, macrophages, natural killer cells, monocytes, T cells, and B cells (**Figure 1B**).

### 3.2 Deconvolution methods validation on simulated and PitNET pseudobulk RNA-seq

Using the snRNA-seq reference established above, we systematically evaluated four deconvolution methods – grouped into marker-based and snRNA-seq-based approaches – to determine the most effective strategy for resolving tumor composition and detecting residual normal tissue.

**Figure 2:**
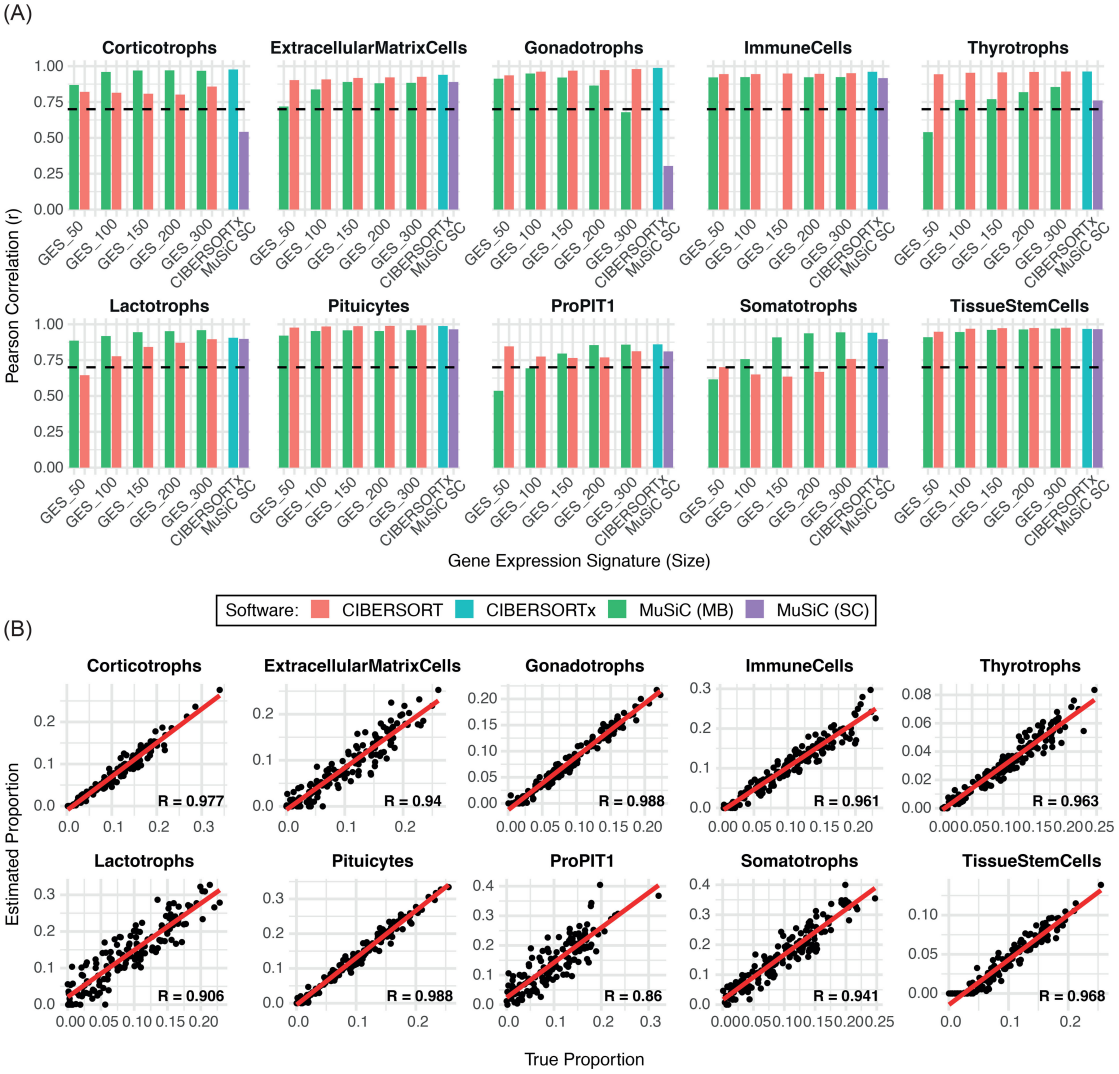
Comparison of the performance of deconvolution methods in estimating individual cell types. **(A)** Bar plot showing the Pearson correlation coefficients (r) between the estimated and true cell-type proportions across 150 simulated samples for each deconvolution method. The dashed line indicates the limit performance threshold (r = 0.70). **(B)** Scatter plots displaying the correlation between estimated and true proportions for each cell population using CIBERSORTx. MC: marker-based SC: single-cell based.

For marker-based deconvolution, cell-type-specific GES were derived from snRNA-seq data (**Supplementary Table 2**) and applied to deconvolute both simulated and PitNET-derived pseudobulk datasets. CIBERSORT and MuSiC showed comparable overall performance across the different GES sizes, with optimal results observed for GES300 (CIBERSORT) and GES200 (MuSiC), respectively (**Figure 2A**, **Supplementary Figure 2A-B**). Notably, despite achieving high overall correlation, CIBERSORT demonstrated reduced accuracy in detecting corticotroph cells (**Supplementary Figure 2A**). In contrast, MuSiC achieved r > 0.8 across all major cell types in the simulated dataset, although its performance decreased for populations representing <5% of total cells (**Supplementary Figure 2B**). In the analysis of 23 PitNET-derived pseudobulk samples, CIBERSORT showed greater precision in detecting tumor cell populations, particularly somatotrophs. However, both methods displayed limited accuracy in characterizing silent and null cell tumors (**Supplementary Table 3**).

For snRNA-seq-based deconvolution, full expression matrices were used without predefined markers. MuSiC exhibited lower sensitivity than CIBERSORTx, particularly for corticotroph (r = 0.54) and gonadotroph cells (r = 0.30), both below the correlation threshold (**Figure 2A**). In contrast, CIBERSORTx demonstrated high sensitivity (r > 0.85) in estimating normal (**Figure 2B**) and PitNET derived samples, except for silent and null tumors (**Supplementary Table 3**). Given its robust performance, CIBERSORTx was applied without pre-grouping cell types into broader categories. The analysis included all cell populations identified from the snRNA-seq dataset, except for dendritic cells, fibroblasts, and B cells, which were excluded due to low representation (fewer than 50 nuclei). CIBERSORTx maintained high sensitivity for most cell types; however, its performance was suboptimal for neurons (r = 0.05), NK cells (r = 0), smooth muscle cells (r = 0.75), and astrocyte-like cells (r = 0.70). Despite correlation values above the minimum threshold for the latter two populations, the corresponding scatter plots showed greater dispersion of data points (**Supplementary Figure 2C**), indicating lower predictive consistency. In tumor samples, these poorly correlated cell types – particularly neurons and smooth muscle cells – were consistently overestimated. This overestimation may have contributed to the underestimation of the pituitary cell component observed across samples (**Supplementary Table 3**).

Based on overall performance in simulated and PitNET-derived datasets, CIBERSORT with GES300 and CIBERSORTx were selected as the optimal approaches for marker-based and single-cell-based deconvolution, respectively.

### 3.3 Cell-type estimation on bulk RNA-seq

To validate the selected deconvolution methods for detecting normal tissue contamination in bulk RNA-seq data, we selected ten surgically resected GH-PitNET from our biobank, pre-characterized by IHC (**Supplementary Table 1**). The cohort included five samples with no evidence of residual normal pituitary tissue, and five samples with confirmed contamination by anterior pituitary remnants. Notably, one of the latter also contained fragments of the posterior pituitary.

**Figure 3:**
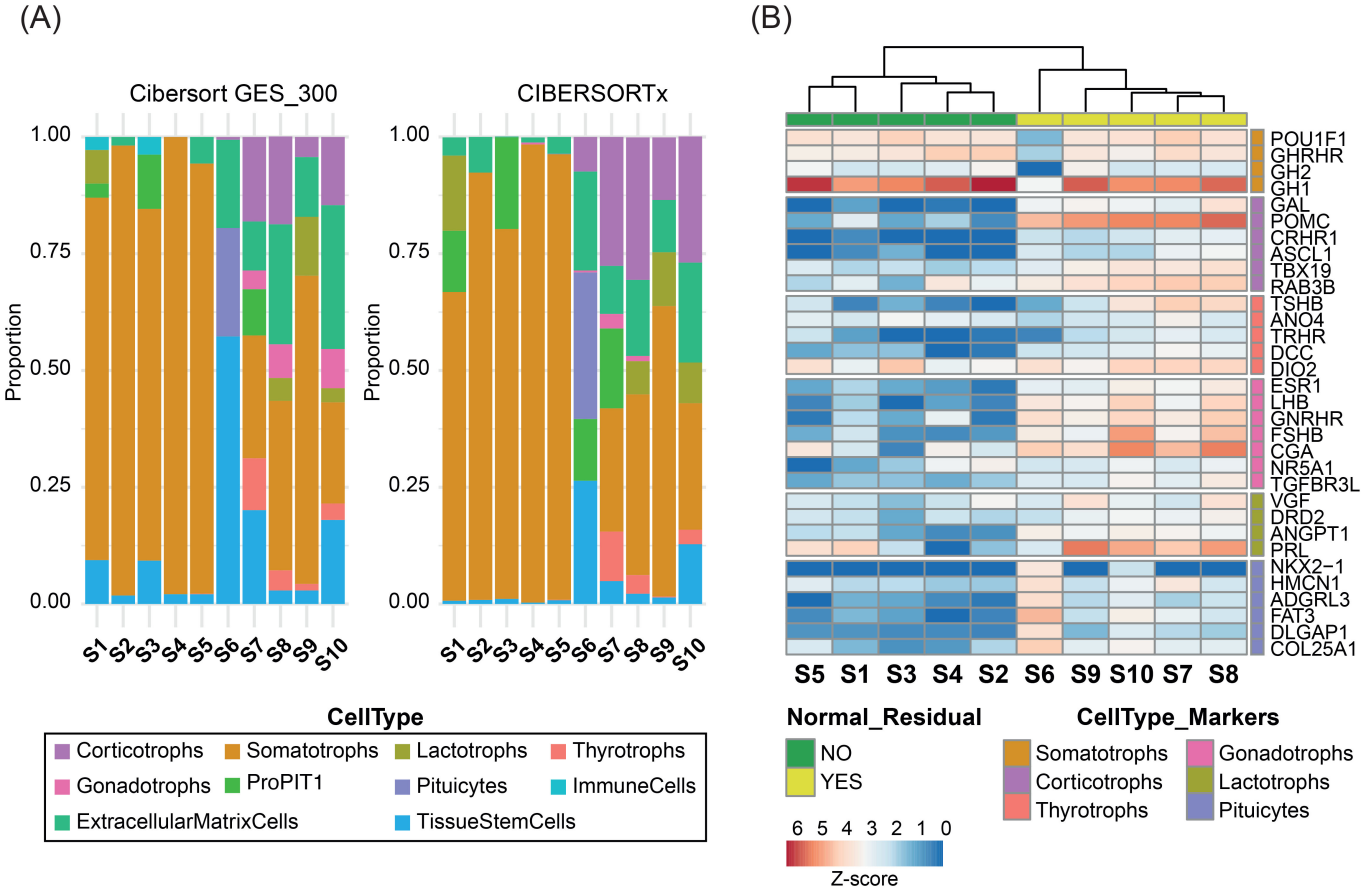
Bulk RNA-seq deconvolution of 10 GH-PitNETs. **(A)** Stacked bar plots representing the estimated proportions of pituitary cell types in 10 GH-PitNETs (5 clean and 5 contaminated), obtained using CIBERSORT with the GES_300 signature (left) and CIBERSORTx (right). **(B)** Heatmap showing Z-score normalized expression of marker genes for each pituitary cell type (as defined in **Supplementary Table 1**) across the 10 GH-PitNETs. Samples were clustered using non-hierarchical clustering. Samples without normal residual tissue by IHC are highlighted in red; contaminated samples are in blue.

Bulk RNA-seq was performed at an average depth of ~131.5 million reads per sample. Transcriptomic deconvolution using the selected best-performing methods consistently identified normal pituitary components in the contaminated samples, in agreement with IHC findings (**Figure 3A**). A heatmap of cell type–specific marker gene expression further supported these observations. The five contaminated samples clustered separately from the uncontaminated tumors and exhibited higher expression of non-somatotroph pituitary lineage markers (**Figure 3B**). Notably, sample S6 showed transcriptomic evidence of posterior pituitary contamination not detected by IHC, with marker gene expression confirming its presence. Conversely, sample S9, identified by IHC as containing posterior pituitary fragments, showed no matching transcriptomic signatures, displaying instead a clear marker gene profile.

To further validate the deconvolution methods across other PitNET subtypes, we analyzed two publicly available bulk RNA-seq datasets encompassing various PitNET subtypes (12, 31). Across both datasets, the selected deconvolution approaches consistently demonstrated robust performance in estimating cell-type proportions within hormonally active tumors. However, their accuracy declined substantially when applied to silent or null cell PitNETs, consistent with previously reported limitations (**Supplementary Table 1**).

**Figure 4:**
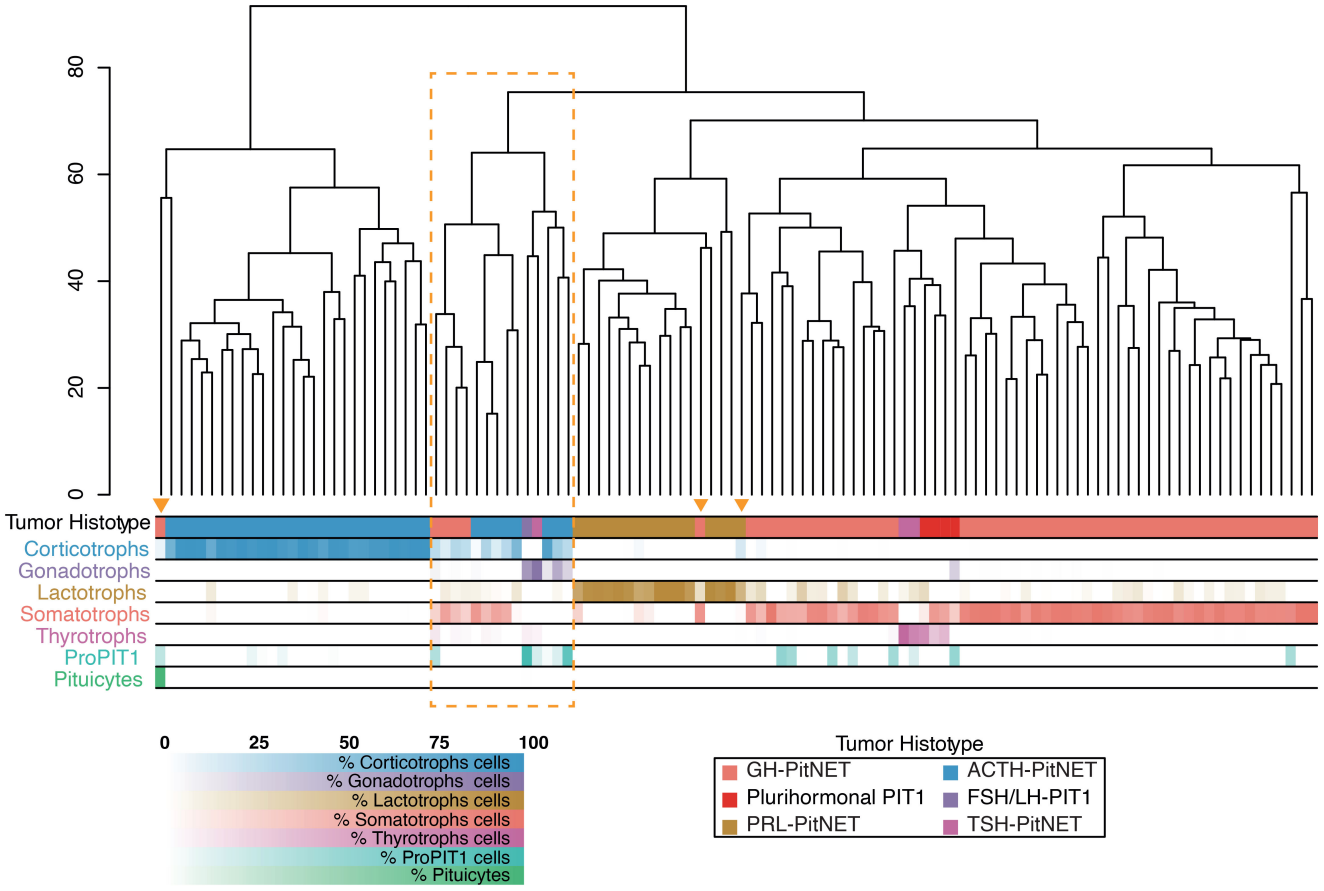
Hierarchical clustering of 114 secreting PitNETs across three integrated datasets. Clustering was performed on the 500 most variable genes after batch correction (distance: Euclidean; linkage: complete). Annotation bars indicate the histological classification of each tumor and the relative proportion of pituitary cell types inferred by CIBERSORTx deconvolution. Samples enclosed in the orange box or marked with a triangle show transcriptomic profiles that diverge from their histological assignment, reflecting contamination from other pituitary lineages.

Despite prior microdissection aimed at enriching neoplastic content in both datasets, we identified additional samples with normal tissue contamination. To assess the transcriptomic impact of contamination, we performed hierarchical clustering of secreting tumors (n = 114), integrating our samples with the two external datasets. Seventeen samples, including our five contaminated ones, consistently clustered either in a distinct group or with tumors of a lineage different from their annotated origin (**Figure 4**). In particular, the residual tissue often belonged to a pituitary lineage distinct from the one indicated by the histotype. According to CIBERSORTx and CIBERSORT, these samples exhibited average contamination levels of 43% ± 19% and 37% ± 22%, respectively.

Eight samples (P002, P003, P0016; P054, P061; P063; P071; P120) had already been annotated as displaying a mixed cellular composition in **Supplementary Table 1** of reference (12), further supporting the presence of mixed histological architecture in these tumors. The remaining samples showed lower average contamination levels – 7% ± 10% with CIBERSORTx and 10% ± 14% with CIBERSORT – mainly due to infiltration by subgroups within the same pituitary lineage. This type of contamination is likely to have a more limited effect on the overall tumor transcriptome.

## 4 Discussion

Bulk RNA-seq has provided valuable insights into the molecular mechanisms and heterogeneity of PitNETs (12–17). However, a major limitation of this approach is its inability to resolve the distinct cellular components within tumor specimens, which frequently include entrapped non-neoplastic pituitary cells (18, 39). Such cellular heterogeneity, if not adequately removed during tissue processing, can distort gene expression profiles and confound the interpretation of tumor-intrinsic transcriptional programs (19).

To overcome this limitation, several computational deconvolution strategies have been developed to estimate the cellular composition of samples analyzed using bulk RNA-seq (26). These approaches rely either on predefined sets of marker genes or on full gene expression matrices derived from scRNA-seq or snRNA-seq datasets, enabling the inference of cell-type contribution to the observed transcriptomic profiles (27, 28, 33). In the present study, we designed and validated a deconvolution framework using different strategies and comparing methods (i.e., CIBERSORT, MuSiC, and CIBERSORTx) leveraging snRNA-seq data from normal anterior pituitary tissue as a reference (26–28). Using this reference, we achieved a highly accurate estimation of cell-type composition in both simulated pseudobulk datasets and PitNET-derived pseudobulk (30), offering a novel perspective on intratumoral complexity and the presence of residual non-neoplastic components. Applying full expression matrices, CIBERSORTx consistently outperformed alternative methods in both sensitivity and accuracy, particularly in hormonally active tumors. Moreover, the use of CIBERSORTx at full resolution – without collapsing cell types into broader categories – enabled the identification of diverse cellular subsets within TME at higher granularity. This approach demonstrated reliable performance across diverse tumor profiles and captured the full spectrum of cellular diversity. MuSiC and CIBERSORT, when applied with curated gene signatures, yielded satisfactory results in simulated datasets. However, MuSiC’s performance markedly declined in PitNET samples, especially when estimating GH-PitNETs, highlighting that CIBERSORT is preferable for marker-based deconvolution. Nevertheless, marker-based tools overall demonstrated limited sensitivity in resolving the diversity of TME cell populations (data not shown), relative to the level of resolution provided by CIBERSORTx.

CIBERSORTx offers greater analytical sensitivity, particularly through batch correction strategies such as B-mode and S-mode, which enhance both the robustness and comparability of deconvolution across datasets (33, 40) Nevertheless, its broader use may be limited by substantial computational demands and reliance on cloud-based infrastructure. In contrast, CIBERSORT can be run locally within the R environment, making it a practical choice for preliminary analyses or use in settings with limited computational resources (27). However, lower resolution and limited ability to accurately capture the cellular diversity of the TME make it less suitable for fine-grained profiling. A recurring limitation across all evaluated deconvolution methods is their reduced reliability when applied to silent or null cell PitNETs – a challenge that is likely attributable to extensive transcriptional alterations affecting key lineage-specific marker genes (12, 31). In such cases, the molecular identity of the tumor may be lost or severely blurred, making conventional deconvolution approaches insufficient for their accurate classification. This highlights the urgent need to identify more specific and lineage-resilient transcriptional markers – a need that, as discussed later, applies not only to these PitNETs subtypes but to all other forms as well. Equally important is the integration of transcriptomic data with complementary modalities, including spatial and proteomic profiling, to achieve a more comprehensive and biologically accurate characterization of tumor identity.

The application of deconvolution methods to our bulk RNA-seq dataset from GH-PitNETs, proved effective in detecting non-tumoral pituitary components in samples previously flagged as contaminated by IHC. In samples S6 and S9, however, transcriptomic and histological assessments yielded partially divergent results regarding the type and extent of pituitary contamination. Since the IHC and RNA-seq analyses were performed on spatially distinct regions of the same specimen, this discordance likely reflects genuine intratumoral heterogeneity, which is part of the complex tissue architecture of PitNETs (41). These findings underscore the importance of integrating post-sequencing quality control strategies to assess both tissue integrity and representativeness, particularly in highly heterogeneous tissues such as PitNETs (42).

To test our approach across different PitNET subtypes, we extended deconvolution analyses to two independent public RNA-seq datasets chosen for their robust experimental design (12, 31). In both studies, tissue microdissection was performed prior to RNA-seq to reduce contamination from residual normal pituitary tissue. Our analysis identified some tumors with non-negligible residual normal pituitary cell content, including all those previously reported as having a heterogeneous composition by Neou et al. (12). Interestingly, clustering analysis of the aggregated dataset revealed that samples with contamination levels exceeding 40% displayed divergent transcriptomic profiles compared to lineage-matched tumors. This effect was especially pronounced when contamination originated from histological lineages distinct from the tumor. Notably, a considerable subset of these samples, drawn from across all three datasets, formed a separate transcriptomic cluster. This apparent distinct molecular entity, however, was clearly a misleading artifact of contamination. The clustering of contaminated samples from independent cohorts validates the robustness of our deconvolution pipeline in consistently resolving biologically confounded profiles. These results underscore the significant risk of misinterpretation in bulk transcriptomic studies when contamination is not properly addressed (43). In this context, deconvolution methods serve not only as essential quality control tools for bulk RNA-seq workflows, but also as effective strategies for detecting hidden non-tumoral components, even in well-processed samples. Moreover, by enabling the detection and exclusion of biologically contaminated samples, deconvolution methods facilitate the integration of heterogeneous datasets within a shared compositional framework. This enhances cross-study comparability based on cell-type representation complementing technical batch adjustment strategies.

The broader applicability of deconvolution methods also has important implications for the design of future transcriptomic studies. Notably, the Neou et al. dataset of microdissected samples shows contamination in approximately 10% of cases. In contrast, our unpublished RNA-seq cohort, which was generated without such refinement, shows contamination in about 20% of cases. These figures offer a useful benchmark for anticipating the impact of tissue heterogeneity and estimating the sample size needed to maintain statistical power in contamination-aware analyses (44).

Beyond contamination-aware analysis, deconvolution also enables deeper investigation of the tumor microenvironment. In this study, we aimed to derive a signature for identifying TME cellular populations, focusing in particular on immune components that critically influence tumor progression across cancers (45). In PitNETs, this area remains underexplored, though recent scRNA-seq studies have begun to provide valuable insights (25, 30, 46). These tumors display a heterogeneous TME (30), with immune composition influenced by tumor hormone secretion: immune cells account for about 3% in functioning tumors and up to 7% in non-functioning ones (30, 47). Among immune cells, macrophages are the most abundant, followed by T cells, and NK cells, while B cells, dendritic cells, neutrophils, and eosinophils are less represented (47, 48). Functionally, lymphoid-dominant immune microenvironments are associated with early recurrence, and T cells tend to be more frequent in invasive tumors (47, 49). Macrophages subsets also interact with tumor cells, modulating growth and invasiveness (25). In this context, CIBERSORTx proved useful for estimating immune cell composition. However, its accuracy was limited by the underrepresentation of stromal and immune cells in the snRNA-seq dataset (29) used to build the reference matrix. This affected the estimation of populations such as dendritic cells, fibroblasts, and B cells, and reduced the precision for identifying neurons, NK cells, and astrocyte-like cells. Nevertheless, the inferred immune infiltration pattern aligned with previous descriptions of pituitary inflammatory microenvironments, predominantly involving macrophages (25, 30), monocytes (50), T cells (30, 46), and capillary endothelial cells. Unfortunately, the low number of immune cells in the dataset prevented the derivation of a specific signature to distinguish activated from non-activated immune states – an area where CIBERSORTx has shown strong performance in other tumor contexts (51).

Despite the clear benefits of applying deconvolution methods to pituitary bulk RNA-seq data, this study also presents notable limitations. A key issue concerns the reference signatures, which were derived exclusively from normal pituitary tissue. While methodologically pragmatic, this choice introduces interpretative bias when analyzing tumor-derived bulk samples. PitNETs often undergo profound transcriptomic remodeling due to oncogenic transformation, hormonal dysregulation, and lineage plasticity. These changes can lead deconvolution algorithms to misassign expression patterns to unrelated cell types, especially when using static, physiology-based reference matrices. A representative example is found in somatotropinomas: several samples showed apparent proPIT1 or lactotroph signatures. These signals likely reflect tumor-intrinsic reprogramming – such as hormone co-secretion or lineage overlap – rather than true contamination by normal cells. This highlights a broader limitation: current deconvolution methods often lack the resolution to distinguish between bona fide residual tissue from neoplastic mimicry, particularly in transcriptionally unstable or mixed-lineage tumors. At present, integrating immunohistochemical data may help validate or refine deconvolution outputs and reduces biases from normal tissue references. However, the lack of tumor-specific single-cell atlases limits the development of tailored reference profiles. Until such resources become available, healthy tissue-based matrices will remain a practical but suboptimal solution for dissecting tumor complexity.

In summary, this study shows that integrating snRNA-seq data with deconvolution methods improves the interpretation of bulk transcriptomic data in PitNETs. Despite limitations related to the biology complexity of pituitary tumors and the incomplete cellular coverage of current single-cell references, this approach may represent a key step toward more precise, reproducible, and clinically meaningful transcriptomic profiling. By clarifying tumor heterogeneity and cellular composition, it may support prognostic assessment and guide patient-specific therapies. Still, its full potential depends on the development of a comprehensive single-cell atlas that includes all tumor subtypes, normal and stromal components, and both functional and non-functional conditions.

## Data Availability

The datasets presented in this study can be found in online repositories. The names of the repository/repositories and accession number(s) can be found below: https://researchdata.cab.unipd.it/1604/, 10.25430/researchdata.cab.unipd.it.00001604.

## Supplementary material

Supplementary Figure 1 Schematic overview of the workflow used for signature derivation, method validation, and application of deconvolution techniques to PitNET datasets.

Supplementary Figure 2 This figure illustrates correlation plots comparing the performance of marker-based and single-cell–based deconvolution methods across normal pituitary and stromal cell populations.

Supplementary Table 1 This table lists the marker genes used to identify cell populations in the snRNA-seq analysis. It also includes immunohistochemistry results from 10 GH-PitNETs. Finally, it reports the deconvolution outputs generated by CIBERSORT and CIBERSORTx for the 10 GH-PitNETs, as well as for samples from Neou et al. and Silva-J. studies.

Supplementary Table 2 This table reports the five generated gene expression signature used as reference for CIBERSORT

Supplementary Table 3 This table reports the true cell-type proportions of the 150 simulated bulk RNA-seq samples, the cell-type proportions obtained from single-cell RNA-seq pro!les of 23 PitNET samples, and the estimated proportions generated by the various deconvolution methods applied to these samples.
