# Supplementary material for: PitNET tissue deconvolution: tracing normal tissue residues and immune dynamics
Source: Front Endocrinol (Lausanne). 2025 Nov 27;16:1674625. doi: 10.3389/fendo.2025.1674625 (PMC12695536; doi:10.3389/fendo.2025.1674625)
Supplement: Supplementary Figure 1 — Schematic overview of the workflow used for signature derivation, method validation, and application of deconvolution techniques to PitNET datasets. [file DataSheet1.pdf]

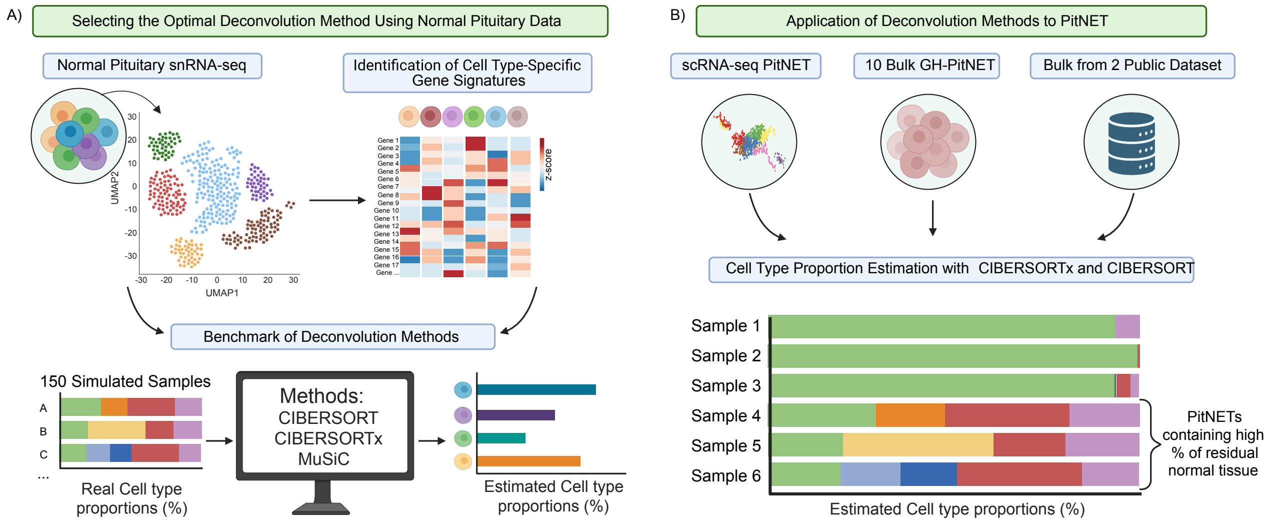

Supplementary Figure 1: Overview of the deconvolution analysis workflow applied to normal pituitary and PitNET data for cell type deconvolution. (A) Single-nucleus RNA sequencing (snRNA-seq) data from normal pituitary tissue were used to identify cell type–specific gene signatures. These signatures were then employed to benchmark deconvolution methods using 150 simulated bulk samples with known cell type proportions, enabling quantitative comparison between estimated and true values. (B) The best-performing methods (CIBERSORT and CIBERSORTx) were applied to PitNET data (including single-cell RNA-seq (scRNA-seq), 10 GH-PitNET, and two public bulk RNA-seq datasets) to estimate cell type proportions and detect residual normal pituitary cells within tumor profiles. Figure created with BioRender.com.
