# Supplementary material for: PitNET tissue deconvolution: tracing normal tissue residues and immune dynamics
Source: Front Endocrinol (Lausanne). 2025 Nov 27;16:1674625. doi: 10.3389/fendo.2025.1674625 (PMC12695536; doi:10.3389/fendo.2025.1674625)
Supplement: Supplementary Figure 2 — This figure illustrates correlation plots comparing the performance of marker-based and single-cell–based deconvolution methods across normal pituitary and stromal cell populations. [file DataSheet2.pdf]

A) Correlation Plots – CIBERSORT_GES300

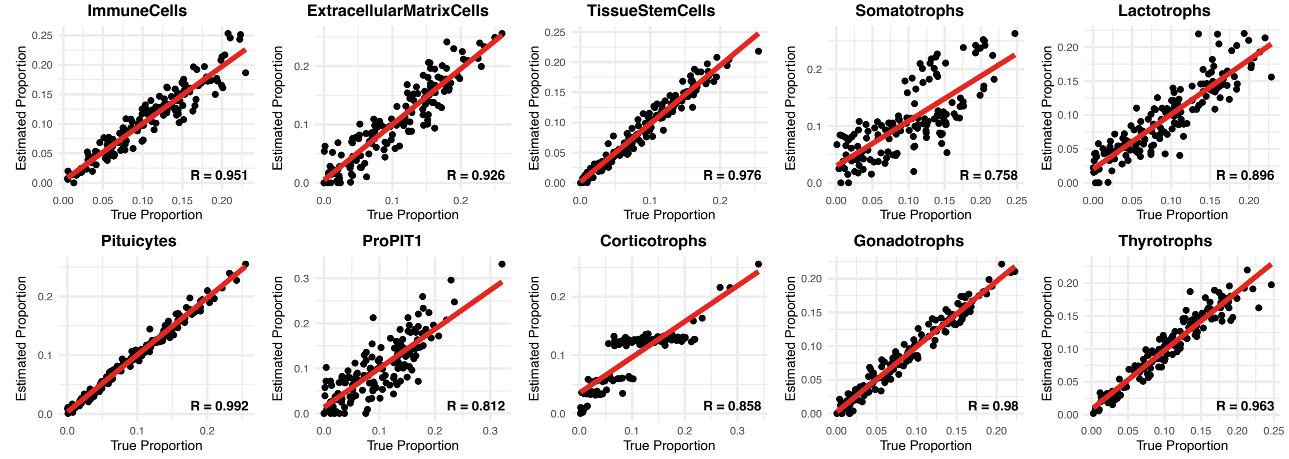

B) Correlation Plots MuSiC – GES200

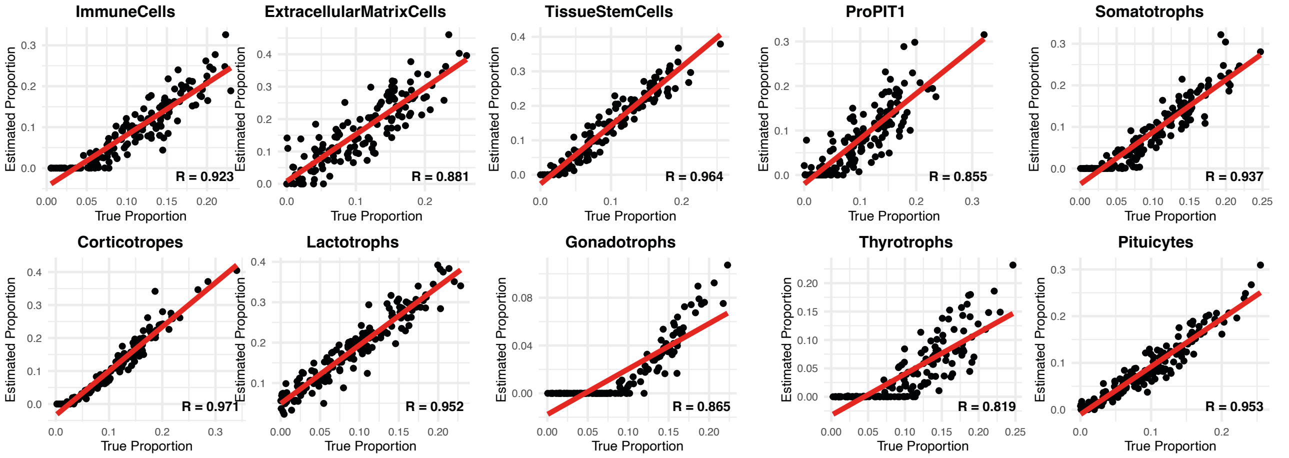

C) Correlation Plots – CIBERSORTX

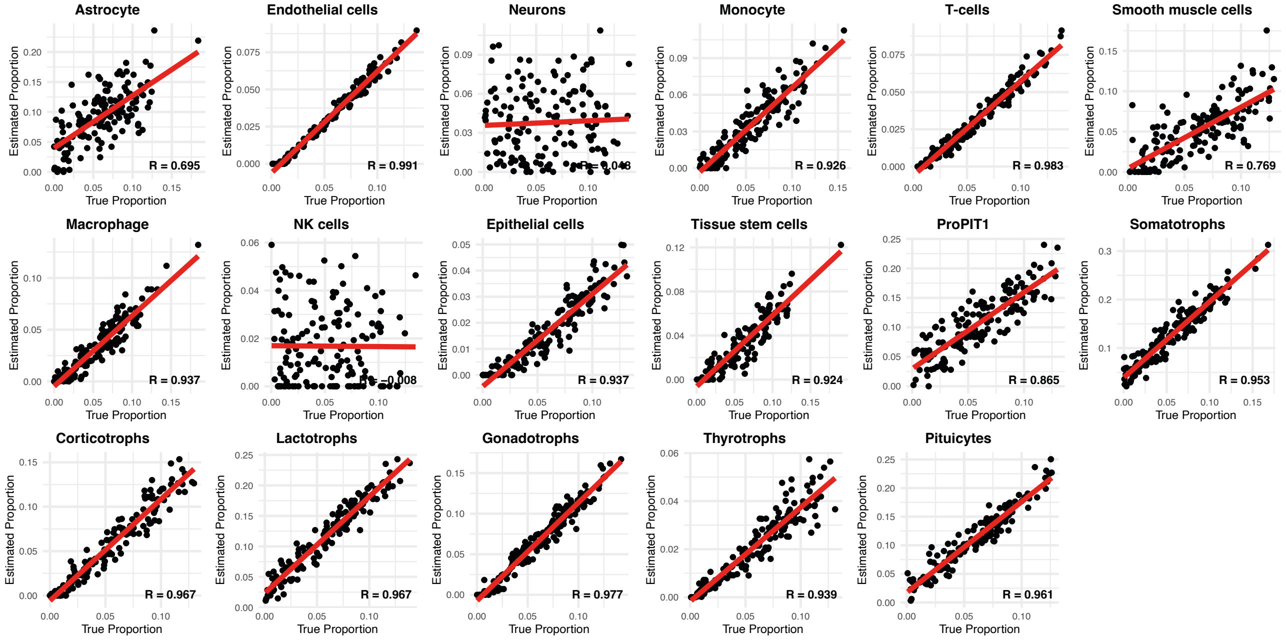

Supplementary Figure 2: Correlation plots comparing marker-based and single-cell–based deconvolution methods applied to normal pituitary and stroma cell populations. Scatter plots display the correlation between estimated and true cell-type proportions across 150 simulated samples. (A, B) Marker-based deconvolution using the best-performing gene expression signatures: (A) GES_300 with CIBERSORT and (B) GES_200 with MuSiC. (C) CIBERSORTx analysis based on snRNA-seq–derived profiles, performed without pre-grouping cell types. Dendritic cells, fibroblasts, and B cells were excluded due to low representation.
